# Challenges and Strategies to Improve Health Literacy in Portuguese-Speaking African Countries

**DOI:** 10.3389/ijph.2024.1606397

**Published:** 2024-04-25

**Authors:** Neida Neto Vicente Ramos

**Affiliations:** Institute of Hygiene and Tropical Medicine, New University of Lisbon, Lisbon, Portugal

**Keywords:** health literacy, Africa, Portuguese-speaking African countries, challenges, strategies

**The IJPH series “Young Researcher Editorial” is a training project of the Swiss School of Public Health.**

Health literacy emerged as a concept in education more than 20 years ago and has since developed dynamically from different perspectives, including personal health literacy and organizational health literacy. Personal health literacy comprises the individual’s ability to obtain, understand, appraise, and apply health information to make appropriate health-related decisions [1]. Organizational health literacy is determined by the extent to which it equitably enables individuals to find, understand, and use information and services to make health-related decisions and take action for themselves and others [1]. Health literacy initiatives are scarce in Portuguese-speaking African countries, and the lack of existing literature suggests a significant deficiency. To explore this critical gap, we highlighted challenges and proposed strategies to improve both personal and organizational health literacy in the Portuguese-speaking African countries (PALOP), which include Angola, Cape Verde, Guinea-Bissau, Mozambique, and São Tomé and Príncipe. These countries were all Portuguese colonies until 1975.

In line with the key recommendations of the World Health Organization, this editorial takes a stance on the vital necessity of strengthening health literacy within PALOP. African governments should put health before political and commercial interests to promote health literacy and build on the excellent recommendations of the World Health Organization (WHO) Global Conferences on Health Promotion [2], particularly the 7th Global Conference on Health Promotion held in Nairobi, Kenya, in 2009 and the 2016 Shanghai Declaration on Health Promotion in the 2030 Agenda for Sustainable Development. Both initiatives emphasized the role of health literacy as a determining factor in supporting capacity building in Africa [2], i.e., empowering individuals and institutions to effectively address challenges, implement sustainable development initiatives, and contribute to the overall progress and wellbeing of the region.

The Community of Portuguese-Speaking Countries (CPLP) and the PALOP have established a collaborative platform that offers a valuable opportunity for these countries to join forces to improve organizational health literacy. By integrating health principles into their local strategies and plans, they can effectively work toward achieving the goals outlined in the 2030 Agenda for Sustainable Development.

Although scientific production is often used to assess the integration of health literacy practices, sub-Saharan African countries produce less than 1% of the world’s research output [3]. Our Medline/Pubmed search in August 2023 for scientific publications on health literacy in local languages (using the terms “health literacy,” “Africa,” and “African countries”) did not yield any articles or books published in PALOP in Portuguese. To reach more readers, some African PALOP authors prefer to publish in English, but doing so becomes a barrier to teaching health literacy to PALOP university students and the public, and slows the dissemination of information to PALOP governments, professionals, and stakeholders. The significance of scientific evidence in shaping organizational health literacy cannot be overstated. It bridges the wealth of medical knowledge generated by researchers with the public’s understanding of health information. Therefore, encouraging the publication of research and health information in Portuguese is crucial for empowering PALOP communities to make informed health decisions.

Like other low- and middle-income countries [4], PALOP must overcome local and structural obstacles that reduce the public’s access to reliable information to meet the challenge of disseminating health messages [5–7]. Much of the population lives in rural areas and has low levels of education. Intermittent access to electricity restricts the population’s use of radio and television; newspapers and magazines have low circulation, and other determinants, including poverty, are associated with low levels of health literacy [6–8].

A two-pronged approach is necessary to address these multifaceted challenges: enhancing personal health literacy and bolstering organizational health literacy. Improving individual health literacy can contribute to more informed health decisions that reduce the consequences of low health literacy, namely, increased hospitalizations and emergency room visits, low adherence to treatment and screening programs, vaccine hesitancy, and poor therapeutic relationships with health professionals [9]. Concurrently, enhancing organizational health literacy requires a concerted effort to ensure that health information and services are accessible and understandable to all [1].

Improving the health literacy of African communities involves the use of tools adapted to local languages, cultures, and technological landscapes. Mobile health initiatives can disseminate health information that addresses maternal health, HIV/AIDS, and preventive care. These initiatives leverage the widespread use of mobile phones in the region. Community radio is an essential source of information in many regions of Africa. It can broadcast health education programs, while local health workshops and community-based health promoters provide face-to-face engagement. Additionally, partnerships with schools and religious institutions can facilitate health education sessions.

In conclusion, this editorial has highlighted the importance of addressing health literacy in PALOP countries and some of the challenges they face in disseminating health information. Prioritizing health literacy in PALOP countries and using the current joint platform established by CPLP to promote it can be a crucial step in empowering organizations, communities, and individuals. A multi-faceted approach that emphasizes collaboration, modernization, investment, and commitment, is key to addressing the need for health literacy initiatives and literature in PALOP. Overcoming the structural and local obstacles that hinder access to reliable health information requires more effective use of existing infrastructure, such as radio and television, and exploring new technologies and platforms that can reach more people, particularly in rural and underserved areas. By focusing on these strategic areas, PALOP can make significant progress in empowering the population with the knowledge and tools necessary for better health outcomes.
